# Broadening the phenotype and genotype spectrum of novel mutations in pontocerebellar hypoplasia with a comprehensive molecular literature review

**DOI:** 10.1186/s12920-024-01810-0

**Published:** 2024-02-13

**Authors:** Mohammad-Reza Ghasemi, Sahand Tehrani Fateh, Aysan Moeinafshar, Hossein Sadeghi, Parvaneh Karimzadeh, Reza Mirfakhraie, Mitra Rezaei, Farzad Hashemi-Gorji, Morteza Rezvani Kashani, Fatemehsadat Fazeli Bavandpour, Saman Bagheri, Parinaz Moghimi, Masoumeh Rostami, Rasoul Madannejad, Hassan Roudgari, Mohammad Miryounesi

**Affiliations:** 1 Department of Medical Genetics, Faculty of Medicine, Shahid Beheshti University of Medical Sciences, , Tehran, Iran; 2 Center for Comprehensive Genetic Services, Shahid Beheshti University of Medical Sciences, Tehran, Iran; 3 School of Medicine, Tehran University of Medical Sciences (TUMS), Tehran, Iran; 4 Pediatric Neurology Department, Mofid Children’s Hospital, School of Medicine, Shahid Beheshti University of Medical Sciences, Tehran, Iran; 5 Genomic Research Center, Shahid Beheshti University of Medical Sciences, Tehran, Iran; 6 School of Medicine, Islamic Azad University Tehran Medical Sciences, Tehran, Iran

**Keywords:** Pontocerebellar Hypoplasia, PCH, Whole exome sequencing, WES, Novel mutations, Novel clinical findings

## Abstract

**Background:**

Pontocerebellar hypoplasia is an umbrella term describing a heterogeneous group of prenatal neurodegenerative disorders mostly affecting the pons and cerebellum, with 17 types associated with 25 genes. However, some types of PCH lack sufficient information, which highlights the importance of investigating and introducing more cases to further elucidate the clinical, radiological, and biochemical features of these disorders. The aim of this study is to provide an in-depth review of PCH and to identify disease genes and their inheritance patterns in 12 distinct Iranian families with clinically confirmed PCH.

**Methods:**

Cases included in this study were selected based on their phenotypic and genetic information available at the Center for Comprehensive Genetic Services. Whole-exome sequencing (WES) was used to discover the underlying genetic etiology of participants' problems, and Sanger sequencing was utilized to confirm any suspected alterations. We also conducted a comprehensive molecular literature review to outline the genetic features of the various subtypes of PCH.

**Results:**

This study classified and described the underlying etiology of PCH into three categories based on the genes involved. Twelve patients also were included, eleven of whom were from consanguineous parents. Ten different variations in 8 genes were found, all of which related to different types of PCH. Six novel variations were reported, including *SEPSECS*, *TSEN2*, *TSEN54*, *AMPD2*, *TOE1*, and *CLP1*. Almost all patients presented with developmental delay, hypotonia, seizure, and microcephaly being common features. Strabismus and elevation in lactate levels in MR spectroscopy were novel phenotypes for the first time in PCH types 7 and 9.

**Conclusions:**

This study merges previously documented phenotypes and genotypes with unique novel ones. Due to the diversity in PCH, we provided guidance for detecting and diagnosing these heterogeneous groups of disorders. Moreover, since certain critical conditions, such as spinal muscular atrophy, can be a differential diagnosis, providing cases with novel variations and clinical findings could further expand the genetic and clinical spectrum of these diseases and help in better diagnosis. Therefore, six novel genetic variants and novel clinical and paraclinical findings have been reported for the first time. Further studies are needed to elucidate the underlying mechanisms and potential therapeutic targets for PCH.

**Supplementary Information:**

The online version contains supplementary material available at 10.1186/s12920-024-01810-0.

## Introduction

Pontocerebellar Hypoplasia (PCH) is a heterogeneous group of rare neurodegenerative disorders that have a fetal onset, which mainly but not exclusively affect the pons and cerebellum. The first report of PCH dates back to 1917; however, the first classification was proposed by Peter G. Barth in 1993. He classified PCH into two types: PCH1, which was defined as degeneration of the spinal cord anterior horn, and PCH2, characterized by chorea/dystonia, microcephaly, severely impaired mental and motor development, and absence of spinal anterior horn pathology [1]. Since 1993, in the following 30 years, many other PCH types have been introduced and added to the OMIM (Online Mendelian Inheritance in Man) database due to significant advances in imaging modalities and genetic sequencing. As of March 16, 2023, OMIM lists (17 types of PCH associated with 25 different genes. PCH1 and PCH2 are the most investigated types, each with six subtypes (A-F). However, some types of PCH are extremely rare, and hence few cases have been reported. For instance, PCH8 has been reported in only three families of Peruvian and Puerto Rican origin [2], PCH10 has been reported in only 11 families of Turkish origin and a family from Sudan [3–5]. Since the first classification of PCH in 1993, its clinical and genetic spectrum has significantly broadened. As mentioned earlier, 17 types of PCH have been introduced, and there is vast inter and intra-heterogeneity among the different types of PCH. Although cerebellum and brainstem development are abnormal in patients with PCH; however, obvious cerebellar symptoms are rarely reported, and symptoms associated with PCH are mostly related to the cortex and basal ganglia dysfunction, including intellectual disability and delayed psychomotor milestones. Although some clinical features may be common between different types of PCH, some specific presentations could help differentiate PCH types, like the disorder of sex development (DSD) in PCH7 [6].

The underlying mechanism of PCH has yet to be understood entirely. Initially, the identification of mutations in the tRNA splicing endonuclease (TSEN) complex led researchers to a hypothesis that mutations in genes involved in tRNA processing (*CLP1, RARS2, SEPSECS, TSEN2, TSEN15, TSEN34, TSEN54*) play a role in PCH etiology. However, subsequent investigations discovered mutations in genes involved in other forms of RNA processing (*EXOSC1, EXOSC3, EXOSC8, EXOSC9, TOE1, PPIL1, PRP17*) and even in genes that were not involved in RNA processing at all (*VRK1, AMPD2, CHMP1A, COASY, MINPP1, PCLO, SLC25A46, TBC1D23, PRDM13, VPS51, VPS53*). As a result, additional functional studies are needed to elucidate the exact etiology of PCH [7, 8].

The scarce information on some types and subtypes of PCH underscores the need for further investigation and the introduction of more cases to better understand the clinical, radiological, and biochemical features of different types of this disease. Moreover, identifying genetic variations in genes related to various PCH types could further expand the genetic spectrum of this disease and aid in the development of focused genetic analysis using a PCH-specific panel. This study presents twelve Iranian probands with novel homozygous variations in PCH-causing genes as well as their clinical and paraclinical presentation. Additionally, a comprehensive literature review of different types of this disease from a molecular perspective is provided.

## Methods and materials

The Center for Comprehensive Genetic Services (CCGS), affiliated with Shahid Beheshti University of Medical Sciences, is a multidisciplinary genetics facility offering patients a range of advanced genetic testing. This facility has conducted numerous genetic tests, totaling in the thousands. The cases encompassed in this study were selected through a retrospective review out of all cases sequenced at the center, with some having been followed up for more than six years. As the Center for Comprehensive Genetic Services as a referral center for patients from all over Iran, it is representative of genetic diseases in the country. All cases with WES reports were screened for homozygous or heterozygous variants in genes related to any type of pontocerebellar hypoplasia. Patients with phenotypes related to any type of pontocerebellar hypoplasia and possible disease-causing variants in pontocerebellar hypoplasia-causing genes were selected. Sanger sequencing was used to confirm the variant in the proband and parents. Cases in which Sanger analysis overruled the variation, were excluded (Supplementary Fig. 1). Ultimately, cases with phenotypes associated with pontocerebellar hypoplasia and genetic variations in related genes were included in this study (Supplementary Fig. 1).

### Sampling and Whole-Exome Sequencing (WES)

The genomic DNA of probands and their parents was extracted from their peripheral blood using the salting out method. The concentration and quality of genomic DNA were assessed by NanoDrop 1000 (Thermo Fisher Scientific, Inc., Wilmington, DE, USA). Whole Exom Sequensing (WES)) was performed on the genomic DNA of probands, using paired-end sequencing on Illumina HiSeq4000, which generates 101-bp paired-end reads. SureSelectXT2 V6 kits were employed to enrich exonic and flanking exon–intron boundary regions.

Burrows-Wheeler Aligner (BWA) was used to map the short reads to the human genome reference (hg19 build) after ensuring the elimination of low-quality reads [9]. SAM tools were used to further process BAM files [10], and Picard was used to remove duplicates (https://broadinstitute.github.io/picard). Then, recalibration and SNP/indel calling were performed. The genome analysis toolkit (GATK) was used for variant calling and filtration based on the best practice [11]. Variant annotation was done using ANNOVAR software. An in-house pipeline was used to annotate, filter, and prioritize the called variants (Supplementary Fig. 2).

### Sanger sequencing

Sanger sequencing was used to confirm the variant found in each proband. For segregation analysis, in order to confirm the variant, it was also checked in the proband's parents. The Sanger sequencing was performed using the BigDye Terminator v3.1 Cycle Sequencing Kit (Life Technologies; Thermo Fisher Scientific, Shanghai, China) on ABI Sequencer 3500XL PE (Applied Biosystems, CA, USA). Polymerase chain reaction (PCR) conditions, purification of the PCR product, and Sanger sequencing were performed based on standard protocols.

## Results

### Demographic

Twelve patients were finally included in this study; four of them were female, and eight of them were male (Table 1). The age at diagnosis spanned from eight months to 4.5 years. All of these families were from Iran, with a high prevalence of consanguinity. Parents were first cousins in cases 1, 5, 7, 10, and 12, second cousins in cases 2, 3, 4, 6, and 9, and third cousins in case 8. The parents of case 11 were not blood-related (Supplementary Fig. 3).

**Table 1:** Genetics, clinical, and MRI findings of cases in this study

Number	Age at dx	Gender	Gene	cDNA change	Protein change	Mutation type	ACMG classification	ACMG Evidence Categories	gnomAD (Agreggated)	ExAC	Mutation Taster	MRI findings	Clinical presentation	OMIM disease
1	8 m	F	*EXOSC3* NM_016042.4	c.395A > C	p.D132A	Missense (Homozygous)	P	PM3, PP1, PS3, PM2, PP3	0.07%	0.03%	Deleterious	Cerebral atrophy, cerebellar atrophy	Hypotonia, hyperreflexia, spasticity, No hearing or visual impairment, neurodevelopmental delay, seizure (died at three years old)	Pontocerebellar hypoplasia, type 1B
2	2 y	M	*EXOSC3* NM_016042.4	c.395A > C	p.D132A	Missense (Homozygous)	P	PM3, PP1, PS3, PM2, PP3	0.07%	0.03%	Deleterious	Cerebellar atrophy	Severe developmental delay, psychomotor regression, mental retardation, Poor head control, speech delay, hypotonia in legs, muscle weakness, spasticity	Pontocerebellar hypoplasia, type 1B
3	10 m	F	*TSEN2* NM_001145394.2	c.749A > G	p.D250G	Missense (Homozygous)	VUS	PM2, BP4	0.0004%	N.A	Benign	N.A	Severe FTT, severe developmental delay, developmental regression (normal up to 4 month), microcephaly, refractory seizure and hypotonia (died at age of 6 years old)	Pontocerebellar hypoplasia type 2B
4	3 y	M	*SEPSECS* NM_006493.4	c.208T > C	p.C70R	Missense (Homozygous)	VUS	PM2, PP3	N.A	N.A	Deleterious	N.A	Truncal hypotonia, mental retardation, developmental delay, delay in walking, speech delay, febrile seizure and strabismus	Pontocerebellar hypoplasia type 2D
5	4 y	M	*SEPSECS* NM_016955.4	c.1274A > G	p.H425R	Missense (Homozygous)	VUS	PM2, PP3	0.0003%	N.A	Deleterious	Cystic cerebellar degeneration	Developmental and motor delay, mental retardation, febrile seizure, spasticity, nystagmus, ataxia and neuropathy (pain sensation)	Pontocerebellar hypoplasia type 2D
6	3.5 y	M	*TSEN54* NM_207346.3	c.1160G > T	p.R387L	Missense (Homozygous)	VUS	PM2	0%	N.A	Deleterious	Atrophy of cerebellum vermis	Meconium aspiration, developmental delay, motor delay, muscle weakness, speech delay, ataxia	Pontocerebellar hypoplasia type 2A/4/5
7	2.5 y	M	*TOE1* NM_025077.4	c.1476C > G	p.F492L	Missense (Homozygous)	VUS	PM2	N.A	N.A	Deleterious	Delayed in white matter myelination	Developmental delay, ambiguous genitalia, strabismus, spasticity, hyperreflexia, microcephaly	Pontocerebellar hypoplasia, type 7
8	1 y	M	*AMPD2* NM_001368809.2	c.1858C > A	p.R620S	Missense (Homozygous)	P	PM2, PM3, PM5, PP2, PP3	N.A	N.A	Deleterious	Periventricular white matter abnormality, elevated lactate level in MRS	Progressive microcephaly, Absent development, seizure, axial hypotonia, spasticity, poor fixation of eye	Pontocerebellar hypoplasia, type 9
9	4 y	F	*CLP1* NM_006831.3	c.784C > G	p.L262V	Missense (Homozygous)	VUS	PM2	0.0005%	N.A	Deleterious	No Abnormal findings in MRI	Motor delay (lack of independent walking), lack of speech, hypotonia, hyperreflexia, epileptic vertigo or dizziness (EVD)	Pontocerebellar hypoplasia, type 10
10	4.5 y	F	*CLP1* NM_006831.3	c.419G > A	p.R140H	Missense (Homozygous)	P	PP1, PP3, PS3, PM2	0.0018%	0.0008%	Deleterious	Cortical atrophy, enlarged ventricular	Poor growth, progressive microcephaly, hypotonia, tonic seizure and developmental and motor delay (lack of independent sitting or walking), lack of speech, scoliosis, sensory neuropathy, strabismus	Pontocerebellar hypoplasia, type 10
11	8 m	M	*CLP1* NM_006831.3	c.419G > A	p.R140H	Missense (Homozygous)	P	PP1, PP3, PS3, PM2	0.0018%	0.0008%	Deleterious	cerebral and cerebellar atrophy, Leukodystrophy	Seizure, developmental delay, progressive microcephaly, hypertonia, spasticity (died at age of 20 months)	Pontocerebellar hypoplasia, type 10
12	13 m	M	*TBC1D23* NM_001199198.3	c.458T > C	p.M153T	Missense (Homozygous)	VUS	PM2	0.0004%	0.0017%	Deleterious	generalized brain atrophy	Delayed psychomotor development, Intellectual disability, language delay, inability to walk, hypotonia (early infancy), muscle atrophy, generalized spasticity, dysphagia, recurrent respiratory infections, happy demeanor, autistic features	Pontocerebellar hypoplasia, type 11

### WES and Sanger sequencing

Using WES, a total read base of 7 million bp was obtained, and after variation calling, around 90,000 variants were detected for each proband. Using an in-house pipeline, these variations were filtered according to American College of Medical Genetics (ACMG) guidelines. Nearly 300 pathogenic, likely pathogenic, and variants of uncertain significance according to ACMG guidelines related to the proband's phenotype were screened by medical geneticists specialized in WES analysis for each proband. In twelve probands that were included in this study, ten different variations in 8 different genes were found, all related to different types of pontocerebellar hypoplasia. Six of these ten variations were novel and had not been reported in databases, including gnomAD and ExAC. These six variants are: *SEPSECS* (c.208T > C:p.C70R), *TSEN2*(c.749A > G:p.D250G), *TSEN54* (c.1160G > T:p.R387L), *AMPD2* (c.1858C > A:p.R620S) *TOE1* (c.1476C > G:p.F492L), and CLP1 (c.784C > G:p.L262V). Two variants in *SEPSECS* (c.1274A > G:p.H425R) and *TBC1D23*(c.458T > C:p.M153T), had been reported in gnomAD or ExAC databases; however, no publications have ever reported the pathogenicity of these variants in pontocerebellar hypoplasia. Two variants in *EXOSC3* (c.395A > C:p.D132A) and; *CLP1*(c.419G > A:p.R140H) had been reported for Pontocerebellar hypoplasia, type 1B and Pontocerebellar hypoplasia, type 10 in literature. Of the ten reported variants in this study, two of them (*EXOSC3*:c.395A > C; *CLP1*:c.419G > A) are pathogenic, one of them is likely pathogenic (*AMPD2*:c.1858C > A), and seven of them are variants of uncertain significance (*SEPSECS*:c.208T > C, *SEPSECS*:c.1274A > G, *TSEN2*:c.749A > G, *TSEN54*:c.1160G > T, *TOE1*:c.1476C > G, *CLP1*:c.784C > G, *TBC1D23*:c.458T > C) according to ACMG guideline. The structure of proteins and the position of mutated amino acids could be found in Supplementary Fig. 4 [12–18].

### Clinical features

Almost all patients presented with developmental delay, although with various severity from lack of independent walking in case 9 to absence of development in case 8. Hypotonia, seizure, and microcephaly are the common features among PCH cases in this study. Magnetic resonance imaging (MRI) reports of almost all cases except for two of them were available, and cerebellar atrophy was the most found feature in cases, followed by cerebral and cortical atrophy. Notably, one of the cases (case 9) had no abnormal MRI findings. The details of clinical and paraclinical findings of each proband can be found in Table 1.

### Classification of PCH based on underlying molecular pathways

Currently, PCH is classified into 17 types, mostly based on the site of the underlying genetic mutation in the genome. Based on the genes involved, the underlying etiology of PCH can be further divided into three groups: tRNA-processing genes (*CLP1, RARS2, SEPSECS, TSEN2, TSEN15, TSEN34, TSEN54*), non-tRNA-processing genes targeting other forms of RNAs (*CDC40, EXOSC1, EXOSC3, EXOSC8, EXOSC9, PTOE1, PPIL1, PRP17*), and genes which are not directly involved in any form of RNA processing (*VRK1, RDM13, AMPD2, CHMP1A, COASY, MINPP1, PCLO, SLC25A46, TBC1D23, VPS51, VPS53*).

### PCH-related genes involved in tRNA-processing

PCH2 subtypes (except for PCH2A, E), PCH4, PCH5, PCH6, and PCH10 are all results of genetic alterations in genes involved in tRNA-processing (detailed clinical presentations of these types of PCH can be found in Table 2). These genes code proteins involved in TSEN protein complex, aminoacyl tRNA synthetase (*RARS2*), or SepSecS enzyme (*SEPSECS*).

**Table 2:** Genetic, clinical, and neuroimaging findings of previously reported PCH cases with genes involved in tRNA-processing

PCH type/subtype	Inheritance	Genetic mutation loci	Phenotypic spectrum												References (PMID)
			Onset	Head and neck	Respiratory	Gastrointestinal	Genitourinary	Soft tissue/Skeletal	Neurologic	Behavioral Psychiatric Manifestations	Endocrine	Paraclinical findings	biochemical	Others	
**PCH2A**	AR	*TSEN54*	at birth	Progressive microcephaly, central visual impairment, abnormal visual pursuit	NR	Poor feeding, Poor sucking	NR	Hypertonia at birth	Profound developmental delay, Restlessness at birth, Inability to sit or control head, Extrapyramidal dyskinesia, Spasticity, Opisthotonus, Seizures	NR	NR	Cerebellar hypoplasia, Pontine hypoplasia, 'Dragonfly-like' pattern, Cortical atrophy, Loss of Purkinje cells, Periventricular white matter abnormalities, Diffuse cerebral gliosis, Absence of transverse pontine fibers	NR	Death in childhood may occur	7854532, 20956791, 20952379
**PCH2B**	AR	*TSEN2*	at birth	Progressive microcephaly, Sloping forehead, Central visual impairment, Lack of visual fixation	NR	Feeding difficulties	NR	Hypotonia	No psychomotor development, Dyskinesias, Dystonia, Clonus, Spasticity, Opisthotonus, Chorea, Axial hypotonia, Limb hypertonia, Extensor plantar responses, Seizures	NR	NR	Cerebellar atrophy, Brainstem hypoplasia, Pontine atrophy, 'Dragonfly' pattern on imaging, Thin corpus callosum, Cerebral atrophy, Ventricular dilatation, Simplified gyral pattern	NR	Death in early childhood may occur	23562994, 20952379
**PCH2C**	AR	*TSEN34*	NR	Central visual impairment	NR	NR	NR	NR	Epileptic seizures	NR	NR	mild involvement of cerebellum and pons	NR	NR	20952379
**PCH2D**	AR	*SEPSECS*	in infancy	Progessive microcephly, ocular nystagmus, head titubation, bilateral optic nerve hypoplasia, visual impairment	progressive chronic respiratory insufficiency	Poor sucking	NR	Contractures in limbs, abolished DTR, Hypotonia	Mental retardation, Lack of psychomotor development, Progressive spastic quadriplegia, Ataxia, Clonus, Seizures, Mild chorea, Sleep disturbances, Bradykinesia, extrapyramidal rigidity, cerebellar syndrome (scanning speech and appendicular dysmetria, with ataxic gait and inability to walk in tandem), pyramidal tract involvement (exaggerated deep tendon reflexes and absent plantar reflex bilaterally)	Irritability	NR	Progressive cerebellar atrophy (cerebellar vermal atrophy before cerebral atrophy), Progressive cerebral atrophy, Delayed myelination, Decreased white matter volume, Thin corpus callosum, Periventricular white matter abnormalities	NR	Reduction in mitochondrial complex I and II activity and an increased number a of type 1 fibers in the muscle (35091508)	25044680, 12920088, 35252561, 35091508, 35637137, 36085396, 29464431, 26888482
**PCH2F**	AR	*TSEN15*	at birth	Progressive microcephaly, Strabismus, Poor or absent eye fixation	NR	NR	NR	Hypotonia	Intellectual disability, Motor delay, Inability to walk, Poor or absent speech, Seizures, Spasticity, Hyperreflexia, Extensor plantar responses	NR	NR	Pontocerebellar hypoplasia, Cortical atrophy	NR	NR	25558065, 27392077
**PCH4**	AR	*TSEN54*	at birth	Microcephaly	Little spontaneous breath, Central respiratory failure	Swallowing disturbances	NR	Hypertonia at birth, Congenital contractures	Profound delayed psychomotor development, Seizures, Spasticity, Myoclonus	NR	NR	Cerebellar hypoplasia, Decreased cerebellar folia, Cerebellar cortex shows normal layers, Loss of Purkinje cells, Pontine hypoplasia, Brainstem hypoplasia, Shrunken inferior olivary nuclei, Neocortical atrophy	NR	Polyhydramnios (prenatal), Death usually in infancy	8480512, 20956791, 20952379, 18711368
**PCH5**	AR	*TSEN54*	in utero	Microcephaly	NR	NR	NR	Congenital contractures	Seizure	NR	NR	Dysplastic C-shaped inferior olivary nuclei, Absent or immature dentate nuclei, Cerebellar cell paucity (more marked in vermis than hemispheres), Cerebellar hypoplasia, Severe olivopontocerebellar hypoplasia	NR	Polyhydramnios (prenatal), Death in neonatal period	16470708
**PCH6**	AR	*RARS2*	at birth	Progressive microcephaly, Dysmorphic features (Bitemporal narrowing, Deep-set eyes, Prominent nasal bridge, Narrow mouth palate), Vision loss, Dysconjugate eye movements	Apneic episodes	Poor sucking, Feeding difficulties	NR	Hypotonia, Edematous hands and feet, Contrature, Reduced activity of mitochondrial respiratory chains	Profound developmental delay, Lack of speech, Poor head control, Seizures, Limb spasticity, Spastic quadriplegia, Clonus, Hyperreflexia	NR	NR	Cerebral atrophy, Cerebellar atrophy, Brainstem atrophy	Increased serum lactate, Increased CSF lactate	Failure to thrive, Death in childhood	20952379, 17847012, 20635367, 25809939, 34717047, 35707589
**PCH10**	AR	*CLP1*	at birth	Progressive microcephaly, Dysmorphic features (Prominent eyes, Long palpebral fissures, High-arched eyebrows, Long eyelashes, Poor eye contact, Esotropia, Strabismus, Nystagmus, Broad nasal root, Hypoplastic alae nasi, Short nose, High-arched palate, Thin upper lip)	NR	NR	Cryptorchidism	Kyphoscoliosis, Hip abnormalities	Profound delayed psychomotor development, Encephalopathy, Lack of independent sitting or walking, Seizures, intractable, Lack of speech, Hypertonia, Spasticity, Hyperreflexia, Axonal sensorimotor neuropathy	NR	NR	Thin corpus callosum, Pontocerebellar hypoplasia, Cortical dysgenesis, Simplified gyral pattern, Cortical atrophy, White matter abnormalities, Enlarged ventricles, Delayed myelination	NR	Poor growth	24766809, 24766810, 29307788

### Mutations in components of the TSEN protein complex

**Fig. 1:**
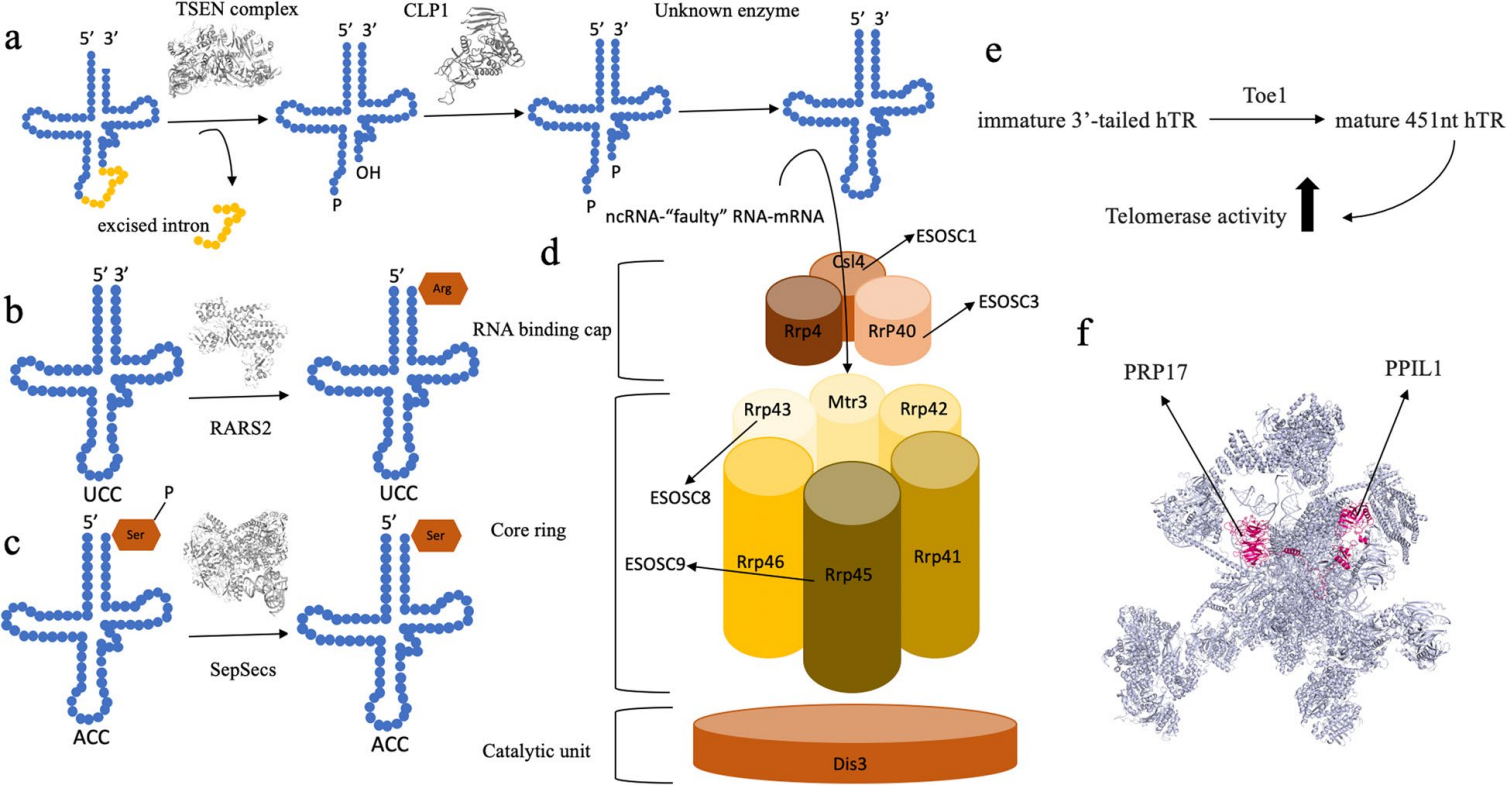
Schematic representation of main pathways involved in PCH. **a** schematic representation of tRNA splicing by TSEN complex and CLP1. **b** charging of Arg-related tRNA by RARS2. **c**) the conversion of O-phosphoseryl-tRNA(Sec) to selenocysteinyl-tRNA by SepSecs. **d**) exosome complex: the structural cap including EXOSC1-3, the core ring including EXOSC4-9, and the catalytic unit made up of DIS3 protein. **e**) Processing of immature 3’-tailed human telomerase RNA component (hTR) to mature 451nt hTR. **f**) structure of the human spliceosome prior to exon ligation. PRP17 and PPIL1 are shown by arrows

tRNAs are RNA subtypes transcribed by RNA polymerase III, involved in protein production in the ribosomal complex. Following transcription, pre-tRNAs undergo a series of post-transcriptional modifications toward becoming mature and functional tRNAs. An important step in this regard is tRNA splicing to remove the intron sections of the transcript. Unlike prokaryotes, Eukaryotic tRNAs do not possess self-splicing qualities and specific splicing enzymatic complexes exist to carry out this role. The tRNA splicing endonuclease (TSEN) complex in eukaryotes, has four subunits TSEN2, TSEN34, TSEN54, and TSEN15, which form a complex along with the regulatory component, CLP1 [19]. The catalytic subunits TSEN2 and TSEN34 are involved in 5´ and 3´ splicing sites’ cleavage. Studies on Archaeal and Eukaryotic TSEN complexes have revealed that the 5´ splicing site requires a motif known as a cation-π sandwich consisting of Arginine 243 and Tryptophan 271 residues at the active site of TSEN34 subunit, and a catalytic triad of Tyrosine, Histidine, and Lysine residues at the active site of TSEN2. Though the 3´splicing site’s cleavage does not need the presence of similar motif on the TSEN2 subunit [20]. Roles of the non-catalytic subunits TSEN15 and TSEN54 as well as the possibly regulatory CLP1 component have not been entirely established and further studies are needed in this regard (Fig. 1a) [19].

PCH2 is presented with signs and symptoms such as developmental retardation, seizure, hypotonia, hypokinesia, visual deficit, and weakness, with a vermis-sparing pattern of cerebellar involvement which leads to a dragon-fly-like pattern on the coronal section of MRI [21]. Similar to PCH1, PCH2 is categorized into six subtypes, PCH2A-F. Four of these subtypes, PCH2A, PCH2B, PCH2C, and PCH2F, result from genetic alterations in members of TSEN family, TSEN54, TSEN2, TSEN34, and TSEN15 respectively [22, 23].

In addition to the aforementioned subtypes of PCH2, PCH4, and PCH5 are also results of genetic mutations in the TSEN gene family member, *TSEN54*. Both types have manifestations such as respiratory impairment, seizure, joint contracture in multiple sites, and clonus [24]. The three types related to *TSEN54* mutations differ in both genetic and MRI findings. PCH2A subtype is a result of a homozygous missense mutation in *TSEN54*, and investigated cases are the result of a change of Alanine 307 residue into a Serine residue. PCH4 cases have compound heterozygous genotypes at the same site or are the result of splice site mutations. PCH5 cases show both heterozygosities at this site and splice site mutations. Such differences in the genetic component of the variants lead to differential findings in imaging modalities, especially MRI. PCH2A abundantly involves disproportional cerebellar hypoplasia with a higher degree of hemisphere involvement and segmentally atrophied cortex, as well as fragmented dentate nucleus and reduction in olivary nuclei folding, reflected in the MRI by a dragonfly pattern. Also, pontine involvement in forms of loss of ventral nuclei and transverse fibers is prominent. PCH4 pathology is differentiated from PCH2 by the absence of foliar structure of vermis, complete loss of both olivary nucleus folding and gliosis along with ventral nuclei and transverse fibers of the pons. MRI findings in this type show microcephalus, pontocerebellar hypoplasia, and retardation of cortical maturation. PCH5 is associated with similar levels of cortical involvement compared to PCH4, though more extensive vermis involvement is prominent, which is also observed in MRI results. This type is also associated with the loss of dentate nuclei in the cerebellum [25].

PCH10 is characterized by microcephalus, developmental retardation, pyramidal manifestations, and mildly atrophied cerebellum. The underlying mutations involve alterations in cleavage factor polyribonucleotide kinase subunit 1 (*CLP1*), a genetic locus encoding a protein involved in tRNA splicing and maturation and 3’ mRNA processing (Fig. 1a) [26, 27].

### *RARS2* genetic mutation

Secondary to the aforementioned post-transcriptional modifications, the tRNA will be ready to get attached to the pertaining amino acid, to get involved in the ribosomal protein synthesis. The enzymatic complex involved in this process are aminoacyl tRNA synthetases (ARS). *RARS2* gene encodes both mitochondrial and cytoplasmic isomers of arginyl tRNA synthetase, which have a role in the attachment of the Arginine residue to the pertaining tRNA during the process of gene expression (Fig. 1b) [28]. The enzyme recognizes both D-loop and anticodon structures of the tRNA and forms an induced fit through conformational changes at the responsive site which at last induces conformational changes in the substrate tRNA and the active site’s structure. Also, the adhesion of the Arginine molecule to the active site helps to maintain the conformational integrity via appropriate positioning of the CCA sequence at the 3´end of the tRNA strand. A variety of key amino acid residues exist in every step of this process [29].

PCH6 is associated with genetic alterations of the mitochondrial arginine tRNA synthetase gene, *RARS2*, and it is characterized by a phenotype of severe epilepsy with early occurrence of first episodes, epileptic encephalopathy, widely distributed brain atrophy, especially in pontocerebellar regions, lactic acidosis, and mitochondrial respiratory chain defects [30].

### *SEPSECS* genetic mutation

PCH2D is caused by mutations in the *SEPSECS* gene that encodes SepSecS, an enzyme in the last step of the selenocysteine production pathway that catalyzes the conversion of O-phosphoseryl-tRNA(Sec) to selenocysteinyl-tRNA (Fig. 1c) [31, 32]. This reaction is the only route of selenocysteine biosynthesis in humans. Since mice with neuronal selenoproteins deficiency show cerebellar hypoplasia, it seems selenoproteins play a crucial role in brain development [33]. Selenoproteins are also involved in antioxidant defense, and reduced selenoproteins levels could damage organs with high mitochondrial activity since mitochondria are one the primary sources of oxidative stress in cells [34].

### PCH-related genes involved in other forms of RNA-processing

PCH1 subtypes (with an exception of PCH1A, E), PCH7, PCH14, and PCH15 are the results of genetic mutations in non-tRNA processing loci (detailed clinical presentations of these types of PCH can be found in Table 3). These genes play roles in RNA exome complex, small nuclear RNA (snRNA) processing, and spliceosome complex.

**Table 3:** Genetic, clinical, and neuroimaging findings of previously reported PCH cases with genes involved in other forms of RNA-processing

PCH type/subtype	Inheritance	Genetic mutation loci	Phenotypic spectrum												References (PMID)
			Onset	Head and neck	Respiratory	Gastrointestinal	Genitourinary	Soft tissue / Skeletal	Neurologic	Behavioral / Psychiatric	Endocrine / Hematology	Paraclinical findings	biochemical findings	Others	
**PCH1B**	AR	*EXOSC3*	at birth	Progressive microcephaly, Poor head control, Oculomotor apraxia, Nystagmus, Poor visual attention, Strabismus, Retinal dystrophy, Tongue atrophy, Tongue fasciculations	Respiratory insufficiency	Poor feeding	NR	Joint contractures, Hip dislocation, Foot deformities, Hypotonia, Muscle weakness, Muscle atrophy	Global developmental delay, Lack of motor milestones, Lack of speech, Spasticity, Hyperreflexia, Seizures, Axonal motor neuropathy	NR	NR	Cerebral atrophy, Cerebellar atrophy, Cerebellar cysts, Atrophy of the pons, Loss of cerebellar Purkinje cells, Loss of cerebellar granular cells, Loss of motor neurons in the spinal cord	NR	Early death may occur	11020648, 12731647, 12548734, 22544365, 23883322
**PCH1C**	AR	*EXOSC8*	in first months of life	Visual impairment, Esotropia, Nystagmus, Ophthalmoparesis, Hearing impairment, Poor head control, Dysmetria, Dysdiadochokinesia	Respiratory insufficiency, Respiratory failure	Poor feeding	NR	Severe muscle weakness, Severe muscle atrophy, Contractures, Hypotonia, mitochondrial respiratory chain (MRC) analysis showed deficiencies of complex I and III	Delayed psychomotor development, Spinal muscular atrophy, Spastic tetraparesis	NR	NR	Cerebellar vermis hypoplasia, Cerebellar atrophy, Thin corpus callosum, Cortical atrophy, Immature myelination, Loss of myelin in the cerebral and cerebellar white matter, Loss of myelin in the descending lateral spinal cord tracts	NR	Fatal in infancy, Failure to thrive	24989451, 34210538
**PCH1D**	AR	*EXOSC9*	at birth or in early infancy	Microcephaly, Poor head control, Dysmorphic facial features (Low-set ears, Nystagmus, Impaired pursuit, Poor or absent fixation, Hypertelorism, Epicanthal folds, High-arched palate, Short neck), Hypomimia	Respiratory insufficiency, Recurrent respiratory infections	Poor suckling reflex, Poor feeding, Difficulty swallowing	NR	Joint contractures, Arthrogryposis multiple congenita, Clenched fists, Adducted thumbs, Pes cavo-varus, Generalized severe hypotonia, Fasciculations, Lack of antigravity movements, Neurogenic atrophy seen on skeletal muscle biopsy	Delayed psychomotor development, Poor gross motor development, Inability to hold head, Inability to sit or walk, Absent language, Spasticity, Hyperreflexia, Seizures, Axonal motor neuronopathy, Clonus	NR	NR	Progressive cerebellar atrophy, Cerebellar hypoplasia, Cerebral atrophy, Delayed myelination, Progressive thalami atrophy	NR	Intrauterine growth retardation (IUGR), Failure to thrive, Poor overall growth, Oligohydramnios (prenatal), Decreased fetal movement, Death in childhood may occur	29727687, 30690203, 33040083, 35893425
**PCH1F**	AR	*EXOSC1*	at birth	Microcephaly, Dysmorphic facial features (Tall forehead, Long philtrum, Smooth philtrum, Retrognathia, Strabismus, Telecanthus, Blue sclerae, Depressed nasal bridge, Anteverted nares, Thick vermilion borders of the lips)	NR	NR	NR	NR	Global developmental delay, Hypotonia	NR	NR	Pontocerebellar hypoplasia, Thin corpus callosum, Cerebral atrophy, Delayed myelination, Hyporeflexia (PNS)	NR	Poor overall growth	33463720
**PCH7**	AR	*TOE1*	at birth	Progressive microcephaly, Oculomotor apraxia, Poor fixation and following, Nystagmus, Optic atrophy, Dysmorphic facial features (Micrognathia,Large ears, Epicanthal folds, Depressed nasal bridge, Broad nasal root, Prominent upper lip)	Abnormal breathing pattern, Apneic episodes	NR	Ambiguous genitalia (Male), Micropenis, Lack of gonadal tissue (Male), Testicular regression	Hypotonia, dystonia	NR	Severe delayed psychomotor development, Developmental delay, Seizures, Moderate intellectual disability, Poor or absent speech, Poor spontaneous movements, Spastic paraplegia, Hyperreflexia, Myoclonus	Increased baseline gonadotropins, Functional anorchia	Pontocerebellar hypoplasia, Cerebral atrophy, Thin corpus callosum, Rudimentary white matter, Lack of ependymal cells, Cerebellar neuronal loss	NR	NR	11068172, 21594990, 23686794, 28092684, 36738896, 34716526
**PCH14**	AR	*PPIL1*	at birth	Progressive microcephaly	NR	NR	NR	NR	NR	Poor or absent psychomotor development, Impaired intellectual development, Absent language, Absent social skills, Hypotonia, Spastic quadriplegia, Brisk reflexes, Dystonia, Seizures	NR	Pontocerebellar hypoplasia, Agenesis of the corpus callosum, Myelination defects, Simplified gyral pattern, Brainstem hypoplasia	NR	Early death may occure	33220177
**PCH15**	AR	*PRP17 (CDC40)*	at birth	Progressive microcephaly	NR	NR	NR	NR	NR	Poor or absent psychomotor development, Impaired intellectual development, Absent language, Absent social skills, Hypertonia, Spastic quadriplegia, Brisk reflexes, Seizures	Anemia, Thrombocytopenia	Pontocerebellar hypoplasia, Partial agenesis of the corpus callosum, Brainstem hypoplasia	NR	NR	33220177

### Mutations in components of RNA exosome complex

PCH1, a major differential diagnosis of spinal muscular atrophy (SMA), involves motor neuron degeneration in the anterior spinal horn as well as progressive pontocerebellar lesions. Clinical manifestations of the disease include the visual and auditory sensory deficit, upper and lower motor signs, ataxia, extrapyramidal manifestations, microcephalus, seizure, developmental impairment, and congenital contractures [35]. PCH1 is further categorized into six subtypes, PCH1A-F, based on the gene which the underlying mutation involves.

The RNA exosome is a multi-subunit protein complex comprised of 9 EXOSC subunits and a ribonuclease involved in the degradation and processing of a variety of RNA molecules. The complex can be divided into three modules; the structural cap including EXOSC1-3, the core ring including EXOSC4-9, and the catalytic unit made up of DIS3 protein (Fig. 1d). In the eukaryotic nucleus, the eleventh subunit, EXOSC10, with riboexonuclease properties is present in close proximity to the cap. The RNA targets of this complex include non-coding RNAs (ncRNA) and “faulty” RNAs in the nucleus, and mRNAs and improper RNAs in the cytoplasm [36].

PCH1B comprises approximately 50% of the PCH1 patients and is a result of Exosome component 3 (*EXOSC3*) gene mutation, which is an indicator of a good prognosis. EXOSC3 is involved in mRNA degradation through encoding component 3 in RNA exosome complex [37].

Mutation of another RNA exosome gene, Exosome component 8 (*EXOSC8*), is seen in PCH1C patients. EXOSC8 expression results in the production of the hexameric ring subunit of RNA exosome. PCH1C patients show a phenotype similar to PCH1B with the addition of hypomyelination [36]. Mutations in two other members of mRNA degradation genes, *EXOSC9* and *EXOSC1*, are responsible for the incidence of PCH1D and PCH1F, respectively [38, 39].

### Mutations in TOE1

PCH7 is presented with developmental retardation, truncal hypotonia, limb hypertonia, episodes of seizure, and hyperactive deep tendon reflexes (DTR), in combination with sexual ambiguity. The underlying mutation of this type is in the target of early growth response 1 (TOE1) locus [40]. *TOE1* encodes a protein involved in snRNA processing [41]. TOE1 is a 3´exonuclease abundant in the Cajal bodies of the cellular nuclei. This enzyme is involved in the processing and maturation of the snRNAs via 3´deadenylation [42]. TOE1 also functions in conjunction with Poly(A)-specific ribonuclease (PARN) as a 3′-to-5′ exonuclease in the maturation process of 3’-tailed human telomerase RNA (hTR) component to mature 451nt hTR (Fig. 1e) [43].

### Mutations in components of the spliceosome complex

Mutations in components of spliceosome complex involved in pre-mRNA splicing, peptidyl prolyl isomerase like-1 (*PPIL1*), and pre-RNA processing 17 (*PRP17*) (CDC40), result in the incidence of PCH14 and PCH15, respectively. The major spliceosomal complex comprises eight cyclophilin peptidyl prolyl isomerases (PPIase), two of which are the aforementioned PPIL1 and PRP17, which form a PPIase-substrate pair (Fig. 1f) [8]. PCH14 and PCH15 have neuropathological characteristics of the pontocerebellar, brain stem, and corpus callosal hypoplasia, developmental delay, seizure, hypo/hypertonia, brisk DTR, spastic features, and microcephalus.

### Other underlying etiologies of PCH

And lastly, subtypes PCH1A, PCH1E, PCH2E, and PCH types 3, 8, 9, 11, 12, 13, 16, and 17 underlying genetic mutations involve genetic loci encoding proteins which are not directly involved in any form of RNA processing (detailed clinical presentations of these types of PCH can be found in Table 4).

**Table 4:** Genetic, clinical, and neuroimaging findings of previously reported PCH cases with genes involved in other underlying etiologies

PCH type/subtype	Inheritance	Genetic mutation loci	Phenotypic spectrum												References (PMID)
			Onset	Head and neck	Respiratory	Gastrointestinal	Genitourinary	Soft tissue / Skeletal	Neurologic	Behavioral / Psychiatric	Endocrine / Hematologic	Neroimaging findings	biochemical findings	Others	
**PCH1A**	AR	*VRK1*	Prenatally or at birth	Microcephaly	Respiratory insufficiency	Poor feeding	NR	Congenital contractures, Foot deformities, Muscle weakness, Fasciculations, Hypotonia	Psychomotor retardation, Mental retardation, Ataxia, Hyperreflexia	NR	NR	Spinal cord anterior horn cell degeneration, Pontocerebellar hypoplasia, Hypoplasia of the ventral pons, Neuronal loss in the brainstem, Neuronal loss in basal ganglia, Gliosis in the brainstem, Gliosis in the basal ganglia,	NR	Death in childhood may occur	12548734, 19646678, 8147499
**PCH1E**	AR	*SLC25A46*	at birth	Dysmorphic facial features (Exotropia, Bitemporal narrowing, Upturned nose with bulbous tip, tented upper lip, narrow palate, flat midface), Optic atrophy, Progressive visual impairment, Nystagmus, Rod-cone dysfunction	Respiratory failure	NR	NR	Scoliosis, Pes cavus, Contacture, Hypotonia, Dysarthria, Neurogenic atrophy, Tapered fingers	Developmental delay, Lack of spontaneous movement, No developmental skills acquired, Ataxic gait, Seizures, Sensorimotor neuropathy	NR	NR	Pontocerebellar hypoplasia, Cerebellar atrophy, Mild atrophy of the brainstem, loss of spinal motor neurons	Increased serum lactate	Polyhydramnios (prenatal), Death may occure in the first days or weeks of life	8147499, 27543974, 27390132, 28653766, 26168012, 27543974, 28558379, 28653766, 36578309
**PCH2E**	AR	*VPS53*	in infancy	Progressive microcephaly, Dysmorphic facial features (Bitemporal narrowing, Micrognathia, Prominent earlobes, Epicanthal folds, Strabismus, Short wide nose,) Poor or absent visual tracking, Optic atrophy, Gaze-evoked nystagmus	NR	NR	NR	Hypotonia, Distal limb edema, Joint contractures, Osteoporosis, Scoliosis	Delayed psychomotor development, Lack of developmental milestones, Mental retardation, Absent speech, Irritability, Seizures, Poor spontaneous movement, Progressive spastic quadriplegia, Opisthotonus, Spasticity	NR	NR	Progressive cerebellar atrophy, Progressive cerebral atrophy, Thin corpus callosum	NR	Short stature, Failure to thrive, Poor overall growth, Progressive disorder	24577744, 12920088, 30100179
**PCH3**	AR	*PCLO*	at birth	Microcephaly, Brachycephaly, Dysmorphic facial features (Long philtrum, Full cheeks, Low-set ears, Large ears, Prominent eyes, Wide palpebral fissures, Depressed nasal bridge, High arched mouth palate), Hearing impairment, Optic atrophy	NR	NR	NR	NR	Developmental delay, Neonatal hypotonia, Poor head control, Seizures, Hyperreflexia, Truncal hypotonia, Spasticity	NR	NR	Small brainstem, Small cerebellum, Cerebral atrophy, Hypoplasia of the corpus callosum,	NR	Short stature, Low weight, Progressive disorder	19277761
**PCH8**	AR	*CHMP1A*	at birth	Microcephaly, Dysmorphic features, Myopia, Astigmatism, Esotropia, Strabismus, Hyperopia, Nystagmus, Cortical visual impairment, Poor visual tracking	NR	Gastroesophageal reflux, Swallowing difficulties	NR	Hypotonia, Joint contractures, Arthrogryposis, Claw feet, Pes cavus, Equinovarus, Talipes valgus	Delayed psychomotor development, Mental retardation, Poor speech, Lack of speech, Lack of independent walking, Truncal hypotonia, Spasticity, Hyperreflexia, Choreiform movements	NR	NR	Cerebellar hypoplasia, Relative preservation of the cerebellar folia, Brainstem hypoplasia, Reduced cerebral white matter, Thin corpus callosum	NR	Poor postnatal growth	23023333, 36694001
**PCH9**	AR	*AMPD2*	at birth or in early infancy	Progressive microcephaly, Optic atrophy, Cortical blindness, Poor eye fixation, Nystagmus, Strabismus,	NR	NR	NR	NR	Delayed psychomotor development, Absent development, Axial hypotonia, Spasticity, Clonus, Hyperreflexia, Seizures	NR	NR	Pontocerebellar hypoplasia, Thin corpus callosum, Fluid filled posterior fossa, Cerebral cortical atrophy, “Figure 8” appearance of midbrain, Ventricular dilatation, Hypomyelination	NR	NR	23911318, 27066553, 29463858
**PCH11**	AR	*TBC1D23*	in early infancy	Microcephaly, Dysmorphic features (Large ears, Strabismus, Esotropia, Hyperopia, Bulbous nasal tip), Poor eye contact, Coloboma, Prominent incisors teeth	Recurrent respiratory infections	Dysphagia	NR	Hypotonia, Muscle atrophy, Talipes equinovarus	Delayed psychomotor development, Intellectual disability, Language delay, Difficulty walking, Cerebellar ataxia, Inability to walk, Wide-based gait, Dysarthria, Poor coordination, Limb ataxia, hyporeflexia of the lower extremities, Spasticity, Seizures	Happy demeanor, Autistic features, Stereotypic behavior, Attention deficit-hyperactivity, Aggressive and auto-aggressive behavior	NR	Hypoplastic corpus callosum, Cortical hypoplasia, Cerebellar atrophy	NR	Short stature, Low weight, Poor overall growth,	28823706, 28823707, 36076253, 32360255
**PCH12**	AR	*COASY*	in utero	Microcephaly, Sloping forehead, Micrognathia	NR	poor sucking	NR	Contractures, Arthrogryposis	Seizures, Spasticity	NR	NR	Cerebellar hypoplasia, Brainstem hypoplasia, Spinal cord hypoplasia, Small cerebrum, Corpus callosum agenesis, Simplified gyral pattern, Optic neuropathy	NR	Polyhydramnios, Death in infancy	30089828, 35499143, 32410094
**PCH13**	AR	*VPS51*	in infancy	Microcephaly,Brachycephaly, Dysmorphic facial features (Hypotonic facies, Full cheeks, Short philtrum, Overfolded ears, Epicanthal folds, Strabismus, Ptosis, Long eyelashes, Nystagmus, Hypertelorism, Upturned nasal tip, Thin upper lip, Thick vermilion of the upper lip, Narrow palate, High-arched palate), Cortical visual impairment, Dental caries	Recurrent respiratory infections, Sleep apnea, Asthma	Feeding difficulties, Tube feeding, Constipation, Cholestatic hepatitis, Hepatomegaly, Hepatic dysfunction	NR	Hypotonia, Lower extremity edema, Pes planus	Global developmental delay, Impaired intellectual development, Absent speech, Inability to sit or walk, Delayed walking, Ataxic gait, Seizures	NR	NR	Cerebellar atrophy, Thin corpus callosum, Cerebral atrophy, Dandy-Walker variant, Periventricular white matter abnormalities, Reduced white matter volume	Abnormal liver enzymes, Hypoglycosylation of serum transferrin	Failure to thrive, Poor overall growth	30624672, 31207318
**PCH16**	AR	*MINPP1*	at birth	Progressive microcephaly, Micrognathia, Low-sets ears, Prominent nose, Nystagmus, Ptosis, Optic atrophy, Cataracts, Abnormal ocular movements, Blindness	NR	Dysphagia, Tube feeding	NR	Scoliosis	Lack of developmental milestones, Lack of independent walking, Delayed motor development, Impaired intellectual development, Poor or absent speech, Poor social interaction, Seizures, Hypotonia (axial), Hypertonia (limb), Spastic tetraplegia, Stereotypic movements, Extrapyramidal signs, Ataxia, Stiffness, Spasticity	NR	NR	Pontocerebellar hypoplasia, Basal ganglia hypoplasia, Thalamic hypoplasia, Thin corpus callosum, Cerebral cortical atrophy	NR	NR	33257696, 33168985
**PCH17**	AR	*PRDM13*	in utero	Microcephaly, Dysmorphic facial features, Visual defects, Cleft palate	Respiratory insufficiency, Apnea, Hypoventilation	Feeding difficulties, Swallowing difficulties, Tube feeding	NR	NR	NR	Neonatal hypotonia, Absent developmental progress, Global developmental delay, Impaired intellectual development, Distal hypertonia, Spastic tetraplegia, Seizures, Autonomic dysfunction	NR	Cerebellar hypoplasia, Brainstem hypoplasia	Hypoglycemia	Poor overall growth, Bradycardia, Hypertension	35390279

### Mutations in VRK1

PCH1A is a result of a mutation in the Vaccinia related kinase 1 (*VRK1*) locus with clinical manifestations of psychomotor retardation, hypotonia, ataxia, poor feeding, and respiratory insufficiency. VRK1 is a serine-threonine kinase mostly located in the nucleus [44]. VRK1 is involved in a variety of cellular pathways via the phosphorylation of different protein groups such as chromatin proteins, transcription factors, and DNA damage response proteins. Chromatin protein substrates of VRK1 include H3 and H2A histones resulting in regulation of histone modification, chromatin compaction, and regulation of gene expression, as well as hnRNP1, phosphorylation of which causes activation of telomerase. VRK1 role in cellular proliferation and tumorigenesis has been investigated extensively. Among the transcription factors targeted by VRK1, phosphorylation of p53, c-Jun, ATF2, CREB, and Sox-2 activates transcription, which is required for cell cycle progression and proliferation [12]. VRK1 deficiency has been shown to cause both developmental and degenerative neurological manifestations. These phenotypes could be due to the disruption of the VRK1/p53 autoregulatory loop that plays a crucial role in cell division and death during nervous system development. On the other hand, since VRK1 activates CREB, mutations in VRK1 may cause neurological phenotypes by disrupting the CREB signaling pathway, as had been shown mutations in RSK2 (CREB kinase) and CREBBP (CREB- binding protein) cause neurological diseases Coffin Lowry syndrome and Rubinstein Taybi syndrome, respectively [12].

### *SLC25A46* mutations

PCH1E is a result of genetic mutations in the Solute carrier family 25 member 46 (*SLC25A46*). *SLC25A46* encodes a protein located in the outer mitochondrion membrane, involved in mitochondrial fission and fusion, maintaining crista structure and facilitation of phospholipid transfer from the endoplasmic reticulum [45, 46]. SLC25A46 is a member of a genetic family encoding mitochondrial carriers, SLC25, encoding transmembrane proteins constructed of three domains, each containing two transmembrane alpha helices connected with a loop at the matrix side of the membrane, all involved in the transportation of a variety of solutes across the mitochondrial membrane [47]. SLC25A46 was first identified in 2006 as a member of the SLC25 family with mitochondrial solute carrier functions widely present in the central nervous system [48]. Knockdown of slc25a46 expression in zebrafish embryos led to brain malformation, spinal motor neuron loss, and poor motility, additionally, studies have shown the balance between mitochondrial fission and fusion is important in cerebellar development and degeneration [46]. Hence mutations in the *SLC25A46* gene could cause a lethal form of PCH with cerebellar atrophy. *SLC25A46* mutations are also associated with a variety of diseases in addition to the lethal PCH1, such as Leigh syndrome and optic atrophy [45, 46].

### Mutations in components of the vesicular trafficking system

The Golgi apparatus is an important subcellular organelle involved in the processing, packaging, and sorting of both secretory and membrane protein structures. Based on a model described as “cisternal maturation”, the newly produced proteins from the endoplasmic reticulum, enter the Golgi apparatus through the *cis*-compartment and undergo several maturation processes towards the *trans* compartment. Meanwhile, retrograde vesicular transportation occurs from *trans* to *cis* compartments in order to recycle the Golgi enzymatic complexes to maintain the localization of such proteins. GARP is a protein complex located at the *trans* compartment of the Golgi complex, comprised of four subunits vascular protein sorting 51 (VPS51), VPS52, VPS53, and VPS54, involved in tethering the endosome-derived vesicles in the aforementioned retrograde trafficking. Subunits VPS51-53, along with another subunit, VPS50, construct a complex with similar functions, endosome-associated recycling protein (EARP) [49].

PCH2E is caused by variants of mutant *VPS53*. The clinical manifestations of the disease include developmental delay, spasticity features, seizure, microcephaly, optic atrophy and nystagmus, and facial dysmorphism [50, 51].

In addition to PCH2E, PCH13 is also a result of mutations in another member of the GARP complex, VPS51. The neuropathological findings of this type include pontocerebellar hypoplasia, developmental impairment, epilepsy, hypotonia, and visual impairment [52].

Another example of eukaryotic vesicular trafficking is the process of synaptic transmission via the release of neurotransmitter-containing vesicles. Through this process, the synaptic vesicles are transferred from the reserved pool to the readily releasable pool at the presynaptic nerve, followed by exocytosis and endocytosis of the vesicle. The filamentous (F)-actin is an important modulator of these steps by means of maintaining the reservoir, transferring the vesicles from this pool, and regulating exocytosis and endocytosis, in contribution to a wide range of proteins. The active zone cytomatrix (CAZ), which F-actin is a part of, is a synaptic structure in association with the release site of the vesicles. F-actin is associated with a variety of protein components in CAZ including piccolo (PCLO), neurexins, and Rab3a-interacting molecules. PCLO is the largest protein among the CAZ-associated proteins with a molecular weight of 560kDa and spans across a number of presynaptic domains, scaffolding a variety of regulators of F-actin function [53].

Variants of the *PCLO* gene are found in cases of PCH3. PCH3 is presented with cerebellar vermis and hemispheres hypoplasia, pontine hypoplasia, atrophied cerebral white matter, seizure within the first year of life, hypotonia, and hyperreflexia [54].

The endosomal sorting complexes required for transport (ESCRT) pathway is an important component of mammalian cell vesicular trafficking. The core components of the ESCRT machinery include both early-acting factors, Bro1 protein family, ESCRT-I, and ESCRT-II, and late-acting factors, ESCRT-III and VPS4. The early-acting proteins are involved in the assembly of ESCRT, membrane deformation, and sorting of the cargo. On the other hand, the late-acting components are involved in membrane fission and disassembly of ESCRT. Among the late-acting factors, ESCRT-III is a protein complex assembled into multiple membrane-bound filaments, with important roles in membrane fission and cofactor recruitment [55]. Eight families of ESCRT-III-related proteins are expressed in humans named charged multivesicular body protein (CHMP)1–8 [56].

PCH8 is characterized by dystonia, ataxia, microcephalus, and non-degenerative, non-progressive cerebellar hypoplasia and is associated with mutations in the *CHMP1A* gene. This gene’s product protein is involved in the ESCRT-III complex and also down-regulates the expression of INK4A, which is an inhibitor of stem cell proliferation. Therefore, the mutations in this locus reduce the rate of proliferation in such cell lineages [2].

Similar to the PCH8 type, PCH11 is characterized by non-degenerative pontocerebellar hypoplasia. In addition, the patients show signs of ataxia, psychomotor developmental delay, and microcephalus. This type is a result of genetic mutations in TBC1 domain 23 (TBC1D23) involved in intracellular vesicular trafficking [57, 58]. Similar to the GARP complex and its pertaining subunits, TBC1D23 is involved in the retrograde Golgi vesicular transportation and is a determinant of specificity in endosome-Golgi vesicular transport at the *trans* compartment of the Golgi apparatus [59].

### Mutations in components of the purine synthesis pathway

The purine synthesis pathway is an important metabolic pathway in both nucleic acid synthesis and energy production by the synthesis of GTP and ATP molecules. Purine biosynthesis is done via two pathways; the de novo pathway starts from ribose 5-phosphate and then its conversion to inosine monophosphate (IMP) which in turn is converted into ATP or GTP, and the salvage pathway which starts with hypoxanthine and guanine which will be converted into IMP and GMP respectively, and adenine is salvaged to AMP by adenine phosphoribosyltransferase [60].

Variants of adenosine monophosphate deaminase 2 (AMPD2)-encoding gene are associated with the incidence of the PCH9. AMPD2 has an important role in maintaining the cellular guanine reservoir by metabolizing AMP into IMP. Therefore, the resultant deficiency of this protein component secondary to loss-of-function mutations results in the impairment of cellular protein production as well as adenosine-caused neurotoxicity. Neuropathological findings of PCH9, involve a combination of microcephalus, pontocerebellar, and corpus callosal hypoplasia. In addition, a pathognomonic imaging finding of the “Figure 8” shape of the midbrain is prominent in axial brain imaging modalities. The clinical manifestations of this type include a severe combination of developmental impairment, seizure, and spastic characteristics [61].

### Mutations in Coenzyme A synthase

Coenzyme A is a key metabolite involved in a wide range of metabolic pathways including fatty acid synthesis, oxidation of pyruvate, and regulation of cell cycle and cell death. This metabolite is synthesized from pantothenic acid. CoA synthetase (COASY) is a mitochondrial enzyme mediating the final steps of this metabolic pathway [62]. PCH12 is caused by mutations in the *COASY* gene. This results in clinical features such as microcephaly, pontocerebellar hypoplasia, arthrogryposis, and death, with prenatal onset [63].

### Mutations in MINPP1

This type has been associated with mutations in multiple inositol-polyphosphate phosphatase 1 (*MINPP1*) gene resulting in intracellular accumulation of inositol polyphosphates, especially inositol hexakiphosphate. Inositol polyphosphates are water-soluble molecules involved in a variety of cellular pathways including the calcium ion-releasing actions of signaling molecule inositol-1,4,5-trisphosphate. The most prevalent forms of these metabolites are inositol-1,3,4,5,6-pentakisphosphate (IP5) and inositol hexakisphosphate (IP6) which are precursors of the integral signaling molecules, inositol pyrophosphates. IP6 is also a structural cofactor in the formation of a variety of protein complexes [64]. The buildup of such anionic metabolites results in the chelation of intracellular cations. Such events result in a neuropathological phenotype of pontocerebellar and cerebral cortex hypoplasia, hypoplastic basal ganglia, spastic tetraplegia, axial hypotonia, distal hypertonia, seizure, and developmental delay [65].

### Mutations in PRDM13

The recently described PCH type, PCH17, is associated with genetic mutations in PRDM13, and was first reported by Coolen et, al. in four families with four different variants of PRDM13 in the regions encoding the zinc finger domain, in 2022. The patients were characterized by developmental retardation, abnormal muscle tone, seizure, as well as hypoplasia in inferior olivary nuclei, and dentate nucleus dysplasia [66]. PRDM family are transcriptional modulators by means of histone methyltransferase actions directly or by recruitment of other histone-modifying proteins [67]. PRDM8 has a role in neural circuit formation by regulation of cadherin-11, PRDM12 is involved in sensory neuron perception, and PRDM15 mutations are found in neurodevelopmental impairment syndromes and progressive nephropathy. PRDM13 is a target of PTF1A and a transcriptional regulator involved in neuronal specification, especially in the spinal cord and retina, as well as the differentiation of GABAergic neurons in the cerebellum [66].

## Discussion

This report identified twelve cases of PCH with variants detected by WES and confirmed through Sanger sequencing, six of which had novel variants. These patients were diagnosed with different PCH types. Six novel homozygous missense mutations in the *TOE1* (c.1476C > G; p.F492L), *AMPD2* (c.1858C > A; p.R620S), *CLP1* (c.784C > G; p.L262V), *TSEN54* (c.1160G > T; p.R387L), *TSEN2* (c.749A > G; p.D250G), and *SEPSECS* (c.208T > C; p.C70R) genes were discovered, resulting in changes in the amino acid sequence of the product proteins. These alterations in protein sequence, structure, and function led to both classic and novel phenotypes, both in clinical characteristics and paraclinical findings. More interestingly, the identification of a novel phenotype in PCH type 9, lactate elevation in MR spectroscopy, can aid in the diagnosis and improved management of PCH type 9. These findings can also contribute to the understanding of the underlying molecular mechanisms and pathways involved in PCH, which can provide insights into the pathophysiology of PCH and may lead to the development of targeted therapies in the future.

PCH is a term describing a group of prenatal neurodegenerative disorders primarily affecting the pons and cerebellum, typically presenting with underdevelopment of specific areas of the brain, microcephaly, motor impairment, and mortality in the early years of life [68]. The disease was first described by Brun in 1917 in a report regarding brain development abnormalities [69]. Bouman et al. adopted the term hypoplasia ponto-neocerebellaris to characterize the sparing of the cerebellar vermis in comparison to hemisphere involvement [70]. Brouwer proposed an underlying mechanism of neurodegeneration rather than the initially stated "hypoplasia" a year later, in 1924 [71]. Krause documented clinical features of the disease in a 16-month-old patient who presented with muscular atrophy, swallowing difficulties, spasticity, and myoclonus in 1929 [72].

In 1993, Barth made the first attempt to classify the illness. He divided it into two categories: type 1, in which anterior spinal horn degeneration is observed, and type 2, in which chorea and dystonia are present. According to this classification, type 1 PCH typically manifests with respiratory impairment, motor involvement, and congenital contractures. Type 2 patients, on the other hand, show signs of microcephaly and developmental impairment in both motor and mental status [1]. Currently, PCH is classified into 17 types, primarily based on the site of the location of the underlying genetic mutation in the genome. As thoroughly discussed in the result section, the underlying etiology of PCH can be divided into three groups based on the underlying mechanism: tRNA-processing genes, non-tRNA-processing genes targeting other forms of RNAs, and genes which are not directly involved in any form of RNA processing.

This study presents a case featuring a novel homozygous mutation in TSEN54 (c.1160G > T; p.R387L) and associated clinical manifestations (case 6): developmental delay, motor delay, speech delay, muscle weakness, and ataxia. MRI findings revealed cerebellar vermis atrophy, aligning more closely with the diagnostic criteria for PCH5, where pronounced vermis involvement is evident in MRI scans. The three PCH types linked to TSEN54 mutations exhibit variations in the nature of genetic mutations. The PCH2A subtype arises from a homozygous mutation, specifically the substitution of Alanine 307 with a Serine residue in TSEN54. PCH4 cases either display compound heterozygous genotypes at the same site or result from splice site mutations. In PCH5 cases, both compound heterozygous genotypes at this site and splice site mutations are observed [24, 25].

The identified mutation in our case (homozygous p.R387L) does not align precisely with the genetic basis of the aforementioned three PCH types. Given the incomplete understanding of genotype–phenotype correlations and the capacity of mutations in TSEN54 to manifest as PCH2A, PCH4, or PCH5, further exploration of novel mutations in TSEN54 and their corresponding clinical presentations is imperative for a comprehensive elucidation of the genetic underpinnings of these disorders.

The PCH10 case (case 9), harboring the c.784C > G variant in the *CLP1* gene, displayed an absence of abnormality in MRI findings, diverging from previous cases characterized by cortical and cerebellar atrophy [31, 32]. Notably, this patient exhibited novel signs, including hypotonia and epileptic vertigo or dizziness (EVD). Conversely, the two additional patients diagnosed with PCH10 (case 10 and 11) shared a similar genotype (c.419G > A homozygous mutation) but presented with slightly distinct phenotypic characteristics.

The first patient, a 4.5-years-old female, manifested growth and developmental retardation, microcephaly, sensorimotor and speech impairment, scoliosis, strabismus, tonic seizures, and hypotonia. Imagining findings revealed cortical atrophy and enlarged ventricles. The second case, an eight-months-old male, Experienced seizure, developmental delay, microcephaly, hypertonia, spasticity, and succumbed at the age of 20 months Imaging disclosed cerebral and cerebellar atrophy alongside leukodystrophy. These variations among patients with similar variants underscore the broad spectrum of phenotypic diversity resulting from alteration in both levels of genetic sequence and regulation of gene expression and their pertaining factors. It is worth mentioning that only 11 families of Turkish origin and a family from Sudan [31] have been reported for PCH10 [3–5] in the literature, and these three cases are the first Iranian cases to be reported.

The c.419G > A mutation that causes the substitution of arginine 140 with histidine has been reported in Turkish families. Functional studies demonstrated that although this mutation does not destabilize the protein, it does impair the kinase activity of the CLP1 enzyme, alters the nuclear localization, and reduces its affinity for the TSEN complex, which together impair the tRNA processing [4].

We identified two patients (case 4,5) with novel homozygous variants in SEPSECS (c.208T > C; p.C70R and c.1274A > G; p.H425R), both presenting with developmental and motor delay and intellectual disability. These cases mark the first instances reported in Iran. Notably, both patients exhibited febrile seizures and eye involvement, encompassing strabismus and nystagmus. Previous instances of PCH2D have typically featured intellectual disability, developmental delay, progressive microcephaly, spasticity, and cerebellar atrophy. However, akin to other PCH types, heterogeneity is observed in this subtype [73]. While nystagmus has been reported in one previous case, our findings constitute the second report of this characteristic [74]. Furthermore, we introduce strabismus as a novel finding in case 4.

In this report, we present two cases of PCH1B (case 1,2), both carrying a homozygous mutation in EXOSC3 (c.395A > C; p.D132A). This variant stands out as the most commonly reported mutation in the EXOSC3 gene and is typically associated with milder forms of PCH1. Previous cases with this variant demonstrated developmental delay but lacked respiratory dysfunctions, usually exhibiting a lifespan extending into adulthood [75]. Contrary to these milder phenotypes, our first case (case 1) exhibited severe manifestations, including neurodevelopmental delay, hypotonia, hyperreflexia, seizures, and succumbed at the age of three years. The disease course was similarly severe in the second case (case 2), suggesting potential involvement of other genetic or environmental modifying factors in the pathogenesis of PCH1B.

EXOSC3 comprises three domains: the N-terminal domain, and the RNA-binding S1 and KH domains. The mutation observed in the presented cases (c.395A > C; p.D132A) is located in a loop interconnecting the strands of the S1 domain. The substitution of the hydrophilic and ionic aspartate with the hydrophobic alanine may compromise the folding of this loop, leading to a distorted structure and impairing its interaction with the catalytic subunits EXOSC5 and EXOSC9 of the exosome complex [36]. However, the wide range of clinical manifestations, spanning from mild to severe forms in patients with the p.D132A mutation, remains challenging to elucidate, and the underlying mechanism is yet to be discovered.

The case of PCH7 (case 7) exhibited previously reported characteristics such as developmental delay and sexual ambiguity, along with strabismus that has only been reported in siblings of Chinese origin (compound heterozygous: c.553C > T;p.R185W, c.562G > T;p.V188L) recently [76]. A novel missense homozygous mutation in TOE1 (c.1476C > G; p.F492L) was identified in this proband, classified as a variant of uncertain significance (VUS) following ACMG guidelines. However, considering the clinical findings in this study, this variant could be pathogenic, emphasizing the importance of analyzing genetic variations within the context of clinical manifestations. The reporting of VUS variants in symptomatic patients holds potential benefits, as they could contribute to PCH diagnosis in a clinical setting.

In the case of the PCH type 9 patient (case 8), novel paraclinical characteristics included an elevation in lactate levels in MR spectroscopy. While elevated lactate levels in serum (PCH1E and PCH6) and CSF (PCH6) have been reported, this study represents the first documentation of elevated lactate levels in PCH9. A novel homozygous mutation in the relatively conserved protein-coding region of AMPD2 (c.1858C > A; p.R620S) [77] was identified in this proband, classified as likely pathogenic according to ACMG guidelines, aligning with the clinical findings of this study.

Variants in the AMPD2-encoding gene are associated with the incidence of PCH9. AMPD2 plays a crucial role in maintaining the cellular guanine reservoir by metabolizing AMP into IMP, thereby contributing to energy production through the synthesis of GTP and ATP molecules. Consequently, deficiency in this protein component due to loss-of-function mutations results in impaired cellular protein production and adenosine-induced neurotoxicity. However, the precise mechanism by which AMPD2 disruption leads to elevated lactate levels necessitates further investigation.

### Genetic counseling and pattern recognition approach

Due to the specificity of the described signs and symptoms, a variety of diseases should be considered as a differential diagnosis of PCH, including congenital disorder of glycosylation type Ia, CASK-related disorders, Tubulin defects, mutations in *RELN* and *VLDLR* genes, Walker-Warburg syndrome, Muscle eye brain disease, Fukuyama muscular dystrophy, pediatric-onset spinocerebellar ataxia, SMA, Joubert’s syndrome, and Dandy-Walker malformation. Though similarities can be observed among these diseases, distinct clinical and paraclinical, as well as genetic testing, can be used to differentiate among such disorders [6]. Since the differential diagnosis of these diseases with PCH had been discussed in detail previously [46, 78], we focus on the clinical findings of PCH, which helps in the differentiation of different PCH types and subtypes. Although WES remains a first-tier diagnostic test for patients with PCH-related signs, establishing specific genotype–phenotype relation could help clinicians in diagnosing PCH by checking a single gene or developing a PCH-specific gene panel. As stated earlier, PCH is a heterogeneous group of neurodegenerative disorders with cerebellar and pons hypoplasia, in which some manifestations such as microcephaly and motor and cognitive impairments are present in almost all individuals. However, there are some manifestations that have been reported in specific types or subtypes of PCH and could be used to differentiate among different types of PCH. For instance, the disorder of sex development has been only reported in PCH7 patients. PCH4,5 are the most severe forms of PCH with polyhydramnios and congenital contracture, which could lead to even prenatal death. The neurological finding could also be helpful since the “eight” pattern is pathognomonic for PCH9 patients and the “dragonfly” pattern is seen in PCH2 patients. Genotype–phenotype correlation is most clear in PCH2A patients, where patients with A307S mutation in the *TSEN54* gene have a “dragonfly” pattern, poor feeding, and extrapyramidal movement disorders. In patients with the aforementioned symptoms and neuroimaging findings, prompt testing for A307S mutation is recommended [6]. Increased serum lactate may help in recognizing PCH6 patients; however, it has also been reported in PCH1E. Ethnicity is another factor to consider when dealing with PCH patients. Until recently, PCH10 has been reported only in people of Turkish origin (a family from Sudan, and three Iranian families of this study have also been added.), or in another instance, PCH2E has been only reported in people of Moroccan Jewish origin. Although ethnic background could be helpful, it should bear in mind that PCH is a very rare disease, and underrepresentation or overrepresentation of cases could make bias towards some specific origin. It is worth mentioning the incessant growing literature regarding PCH has expanded the genotypic and phenotypic spectrum of this disease, leading to the introduction of four new types of PCH since 2020. This expansion will probably continue in the incoming years and add more types and subtypes to the PCH disorder group. Regarding heterogeneity in PCH disorders, the diagnostic work-up should be customized, considering the cost–benefit of each patient. In the absence of clinical clues, comprehensive genetic testing like WES or WGS could be beneficial. However, WES or WGS interpretation could be more fruitful when taking clinical, imaging, and laboratory input into consideration. In the case of a patient with clinical suspicion of PCH, a PCH gene panel could be the diagnostic choice; however, in a more specified manner, if the patient has some specific clinical or paraclinical manifestations that point to a specific type of PCH, checking that single gene may be the most beneficial approach. The last approach is only plausible by defining hallmarks for each type and subtype of PCH, which requires more cases [6, 48, 78]. A pattern recognition approach mainly based on imaging was proposed by Rusch et al., in 2020 when 13 types of PCH were listed in the OMIM database. However, four types of PCH have been added to OMIM since then, and due to the overlap of clinical and neuroimaging findings among these different types, genome-wide genetic testing remains the first choice for PCH diagnosis [79].

## Conclusion

In this study, novel and distinct phenotypes and genotypes are combined with previously described information. We offered recommendations for identifying and diagnosing these various subgroups of disorders due to the diversity in PCH. Hence, providing cases with novel variations and clinical findings could further expand the genetic and clinical spectrum of these diseases and help in better diagnosis. This is because certain critical conditions, such as spinal muscular atrophy, are part of their differential diagnosis. Thus, for the first time, six novel genetic variants, as well as novel clinical and paraclinical findings, have been reported. Further studies are needed to elucidate the underlying mechanisms and potential therapeutic targets for PCH. It is, therefore, crucial to continue investigating these novel phenotypes and their implications for PCH diagnosis and treatment.

### Supplementary Information

**Additional file 1: Supplementary Figure 1.** Flowchart of included cases in this study. **Supplementary Figure 2.** Variant filtering and pathogenicity evaluation algorithm. **Supplementary Figure 3.** Pedigree of included cases in this study. Pedigree a-k are cases 1-12, respectively. The proband is shown by an arrow in each pedigree. Circle and squares represent female and male, respectively. People with same color in each pedigree have same clinical manifestations. **Supplementary Figure 4.** The structure of protein [1] included in this study and the position of mutated amino acid. a) Structure of human nuclear RNA exosome (PDB: 6H25) [2]. EXOS3 is shown by an arrow and the position of Asp132 which is substituted with Ala in case 1 and 2 b) Structure human tRNA Splicing Endonuclease (TSEN) Complex (PDB: 7UXA) [3]. TSEN2 and TSEN54 are shown by arrows c) Structure of human holo SepSecS (PDB: 7L1T) [4] and the position of Cys70 and His425 which are substituted with Arg in case 4 and 5 d) Structure of AMP deaminase 2 (PDB: 8HUB)[5] and the position of Arg 620 which is substituted with Ser in case 8 e) Structure of CLP1(Swiss model: Q92989) [6] and the position of Leu262 which is substituted with Val in case 9 and Arg140 which is substituted with His in case 10 and 11 f) Structure of TBC1D23 N terminal domain (PDB: 6JL7) [7] and the position of Met 153 which is substituted with Thr in case 12.

## Data Availability

The data that support the findings of this study are available from the corresponding authors, upon request.

## Abbreviations

ACMG: American college of medical genetics
AMPD2: Adenosine monophosphate deaminase 2
BWA: Burrows-Wheeler Aligner
CAZ: Active zone cytomatrix
CCGS: Center for Comprehensive Genetic Services
CHMP: Charged multivesicular body protein
CLP1: Cleavage factor polyribonucleotide kinase subunit 1
COASY: CoA synthetase
DSD: Disorder of sex development
DTR: Deep tendon reflex
ESCRT: Endosomal sorting complexes required for transport
EXOSC: Exosome component
EVD: Epileptic vertigo or dizziness
GATK: Genome analysis toolkit
hTR: Human telomerase RNA
IMP: Inosine monophosphate
IP6: Inositol hexakisphosphate
MINPP1: Multiple inositol-polyphosphate phosphatase 1
MRI: Magnetic resonance imaging
PCH: Pontocerebellar hypoplasia
PCR: Polymerase chain reaction
PPIase: Peptidyl prolyl isomerases
PPIL1: Peptidyl prolyl isomerase like-1
PRP17: Pre-RNA processing 17
SLC25A46: Solute carrier family 25 member 46
SMA: Spinal muscular atrophy
snRNA: Small nuclear RNA
TBC1D23: TBC1 domain 23
TOE1: Target of early growth response 1
TSEN: TRNA splicing endonuclease
VPS: Vascular protein sorting
VRK1: Vaccinia related kinase 1
VUS: Variant of uncertain significance
WES: Whole exome sequencing
WGS: Whole genome sequencing
