# Supplementary material for: Broadening the phenotype and genotype spectrum of novel mutations in pontocerebellar hypoplasia with a comprehensive molecular literature review
Source: BMC Med Genomics. 2024 Feb 13;17:51. doi: 10.1186/s12920-024-01810-0 (PMC10863249; doi:10.1186/s12920-024-01810-0)
Supplement: Supplementary file 1 — Additional file 1: Supplementary Figure 1. Flowchart of included cases in this study. Supplementary Figure 2. Variant filtering and pathogenicity evaluation algorithm. Supplementary Figure 3. Pedigree of included cases in this study. Pedigree a-k are cases 1-12, respectively. The proband is shown by an arrow in each pedigree. Circle and squares represent female and male, respectively. People with same color in each pedigree have same clinical manifestations. Supplementary Figure 4. The structure of protein [1] included in this study and the position of mutated amino acid. a) Structure of human nuclear RNA exosome (PDB: 6H25) [2]. EXOS3 is shown by an arrow and the position of Asp132 which is substituted with Ala in case 1 and 2 b) Structure human tRNA Splicing Endonuclease (TSEN) Complex (PDB: 7UXA) [3]. TSEN2 and TSEN54 are shown by arrows c) Structure of human holo SepSecS (PDB: 7L1T) [4] and the position of Cys70 and His425 which are substituted with Arg in case 4 and 5 d) Structure of AMP deaminase 2 (PDB: 8HUB)[5] and the position of Arg 620 which is substituted with Ser in case 8 e) Structure of CLP1(Swiss model: Q92989) [6] and the position of Leu262 which is substituted with Val in case 9 and Arg140 which is substituted with His in case 10 and 11 f) Structure of TBC1D23 N terminal domain (PDB: 6JL7) [7] and the position of Met 153 which is substituted with Thr in case 12. [file 12920_2024_1810_MOESM1_ESM.docx]

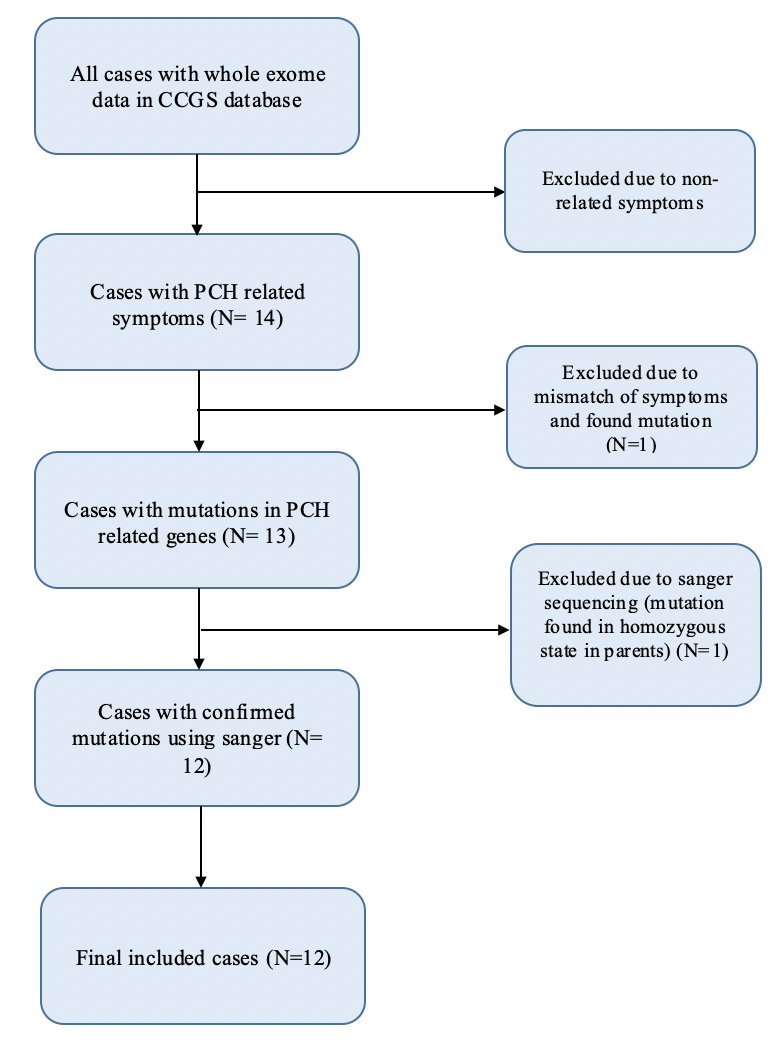


**Supplementary Figure 1.** Flowchart of included cases in this study.

**
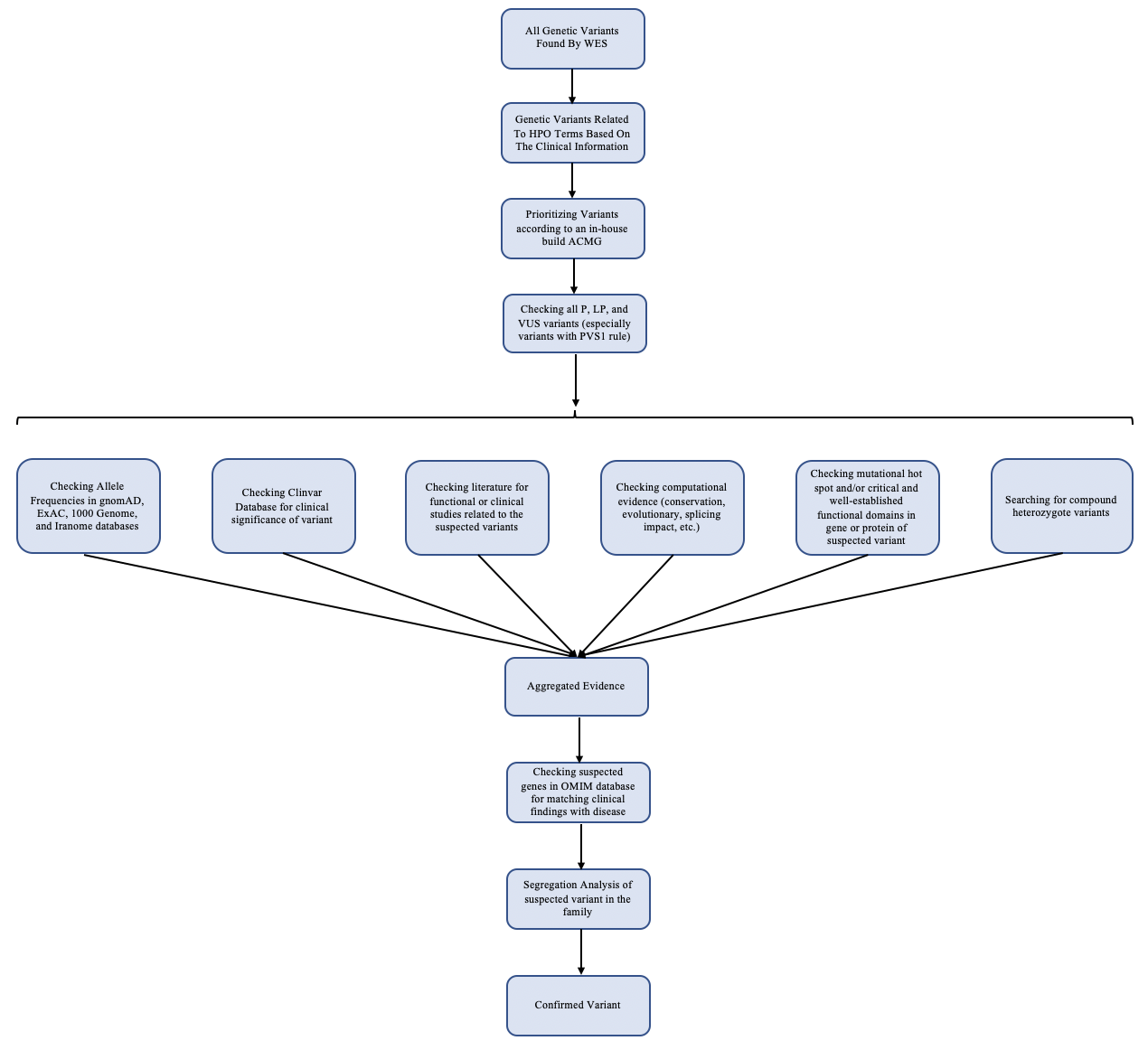
**

**Supplementary Figure 2.** Variant filtering and pathogenicity evaluation algorithm

**Supplementary**
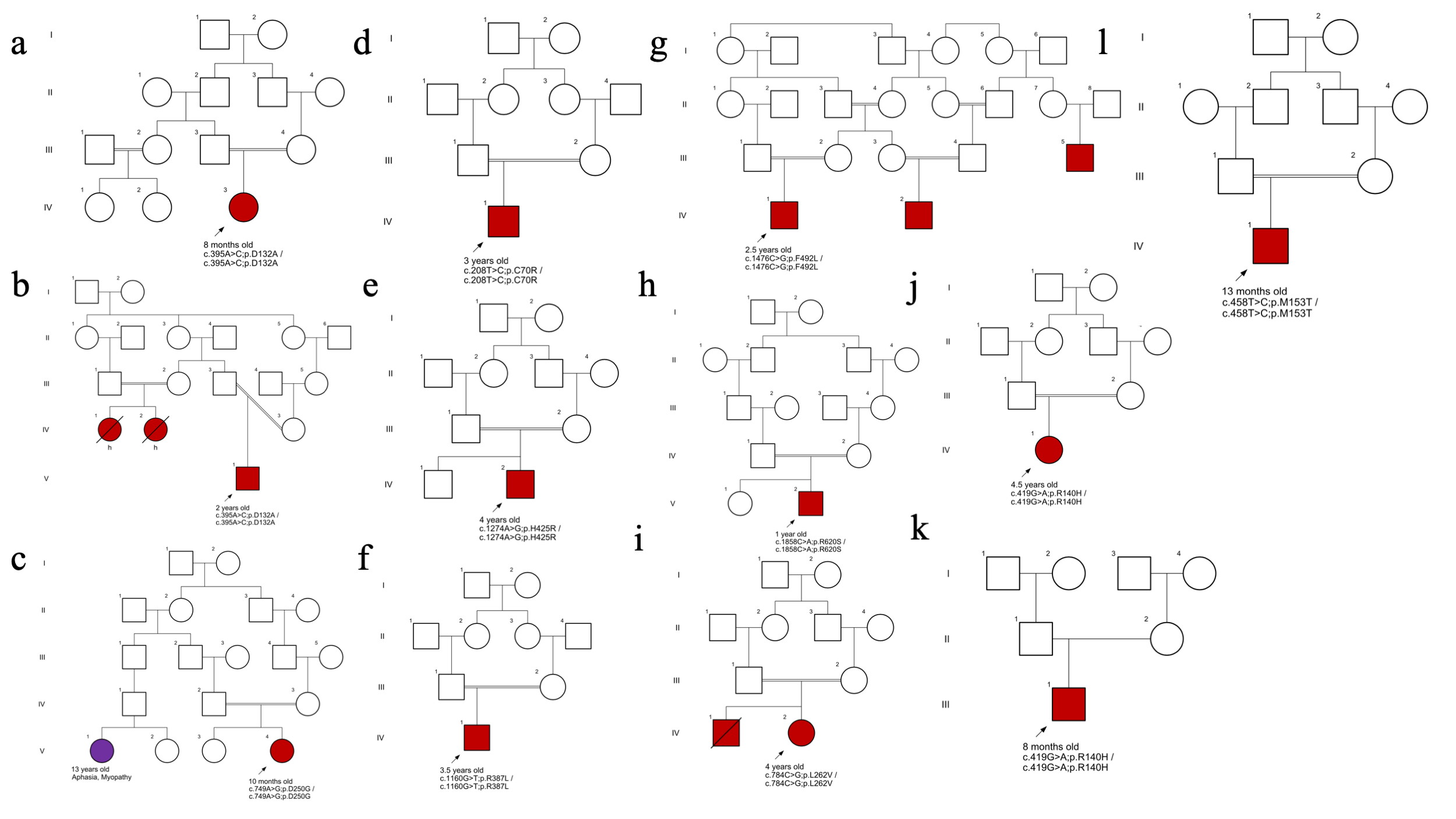
**Figure 3.** Pedigree of included cases in this study. Pedigree a-k are cases 1-12, respectively. The proband is shown by an arrow in each pedigree. Circle and squares represent female and male, respectively. People with same color in each pedigree have same clinical manifestations.


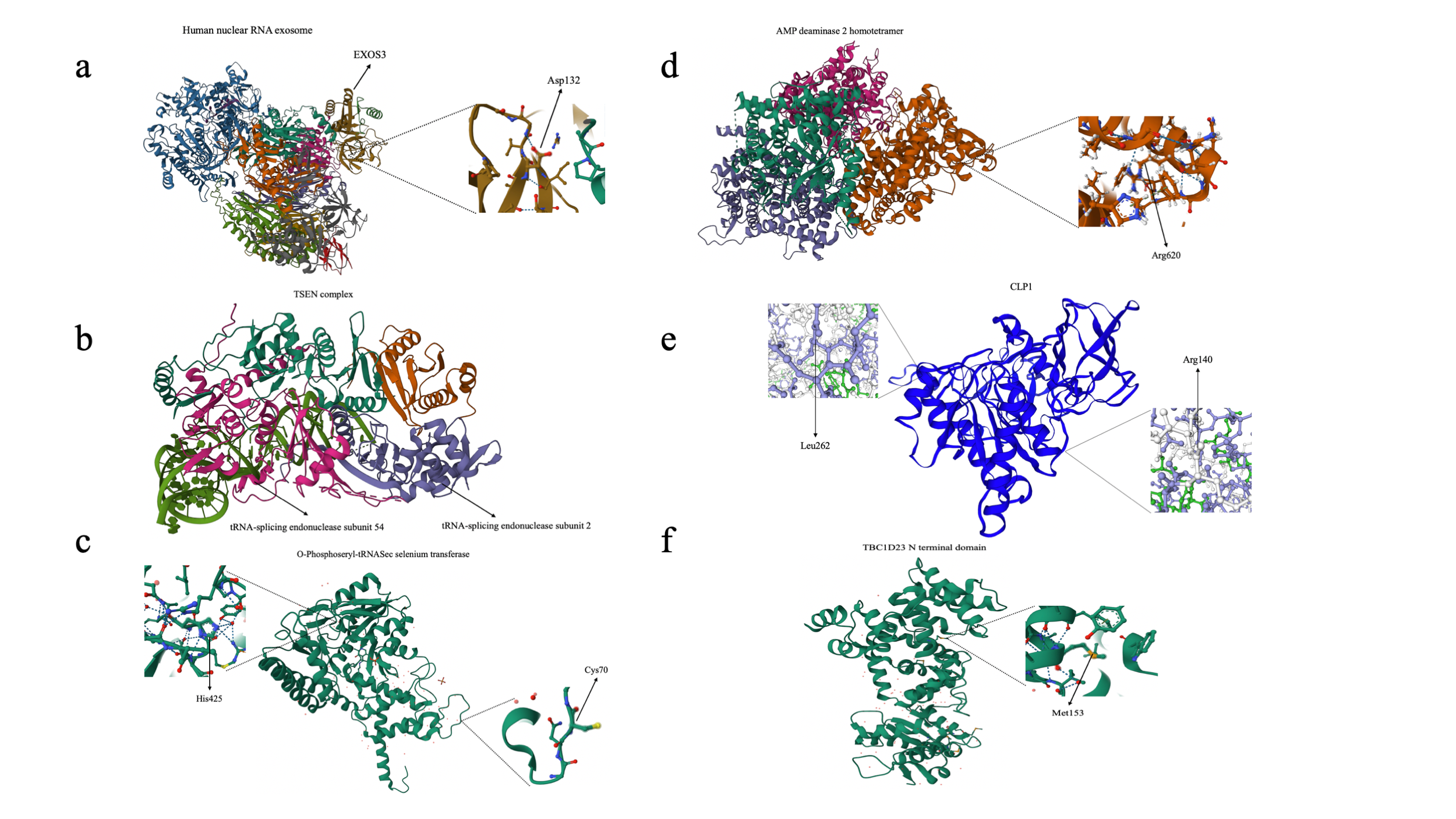


**Supplementary Figure 4.** The structure of protein [1] included in this study and the position of mutated amino acid. **a)** Structure of human nuclear RNA exosome (PDB: 6H25) [2]. EXOS3 is shown by an arrow and the position of Asp132 which is substituted with Ala in case 1 and 2 **b)** Structure human tRNA Splicing Endonuclease (TSEN) Complex (PDB: 7UXA) [3]. TSEN2 and TSEN54 are shown by arrows **c)** Structure of human holo SepSecS (PDB: 7L1T) [4] and the position of Cys70 and His425 which are substituted with Arg in case 4 and 5 d) Structure of AMP deaminase 2 (PDB: 8HUB)[5] and the position of Arg 620 which is substituted with Ser in case 8 e) Structure of CLP1(Swiss model: Q92989) [6] and the position of Leu262 which is substituted with Val in case 9 and Arg140 which is substituted with His in case 10 and 11 f) Structure of TBC1D23 N terminal domain (PDB: 6JL7) [7] and the position of Met 153 which is substituted with Thr in case 12

**References**

1. Berman, H.M., et al., *The protein data bank.* Nucleic acids research, 2000. **28**(1): p. 235-242.

2. Gerlach, P., et al., *Distinct and evolutionary conserved structural features of the human nuclear exosome complex.* Elife, 2018. **7**.

3. Hayne, C.K., et al., *Structural Basis for pre-tRNA Recognition and Processing by the Human tRNA Splicing Endonuclease Complex.* bioRxiv, 2022: p. 2022.09. 02.506201.

4. Puppala, A.K., et al., *Structural basis for the tRNA-dependent activation of the terminal complex of selenocysteine synthesis in humans.* Nucleic Acids Res, 2023.

5. Kitao, Y., et al., *The discovery of 3, 3-dimethyl-1, 2, 3, 4-tetrahydroquinoxaline-1-carboxamides as AMPD2 inhibitors with a novel mechanism of action.* Bioorganic & Medicinal Chemistry Letters, 2023. **80**: p. 129110.

6. Waterhouse, A., et al., *SWISS-MODEL: homology modelling of protein structures and complexes.* Nucleic acids research, 2018. **46**(W1): p. W296-W303.

7. Liu, D., et al., *Structure of TBC1D23 N-terminus reveals a novel role for rhodanese domain.* PLoS Biol, 2020. **18**(5): p. e3000746.
